# Supplementary material for: Causal Relationship between Aging and Anorexia Nervosa: A White-Matter-Microstructure-Mediated Mendelian Randomization Analysis
Source: Biomedicines. 2024 Aug 16;12(8):1874. doi: 10.3390/biomedicines12081874 (PMC11351342; doi:10.3390/biomedicines12081874)
Supplement: Supplementary file 1 [file biomedicines-12-01874-s001.zip › Supplementary Figure/Figure S4/Singe SNP plot of MR analysis for TL to ubm-b-1732.pdf]

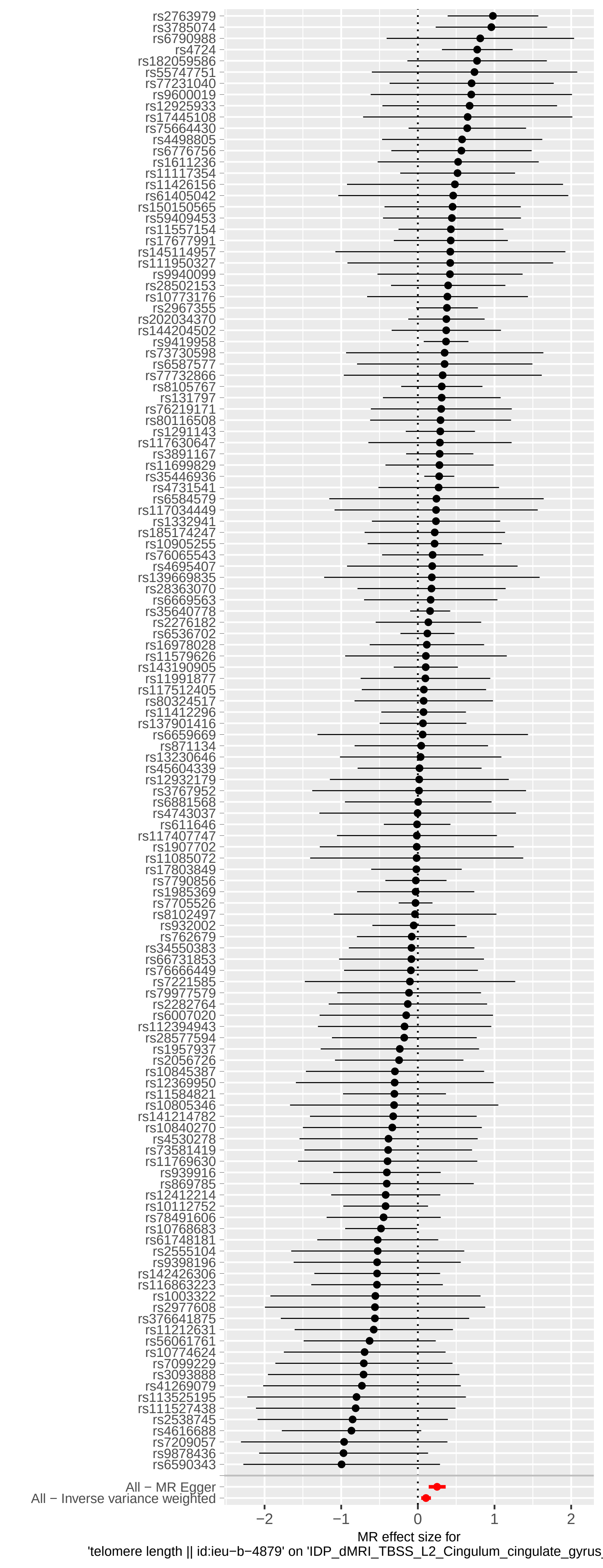
rs2763979
rs3785074
rs6790988
rs4724
rs182059586
rs55747751
rs77231040
rs9600019
rs12925933
rs17445108
rs75664430
rs4498805
rs6776756
rs1611236
rs11117354
rs11426156
rs61405042
rs150150565
rs59409453
rs11557154
rs17677991
rs145114957
rs111950327
rs9940099
rs28502153
rs10773176
rs2967355
rs202034370
rs144204502
rs9419958
rs73730598
rs6587577
rs77732866
rs8105767
rs131797
rs76219171
rs80116508
rs1291143
rs117630647
rs3891167
rs11699829
rs35446936
rs4731541
rs6584579
rs117034449
rs1332941
rs185174247
rs10905255
rs76065543
rs4695407
rs139669835
rs28363070
rs6669563
rs35640778
rs2276182
rs6536702
rs16978028
rs11579626
rs143190905
rs11991877
rs117512405
rs80324517
rs11412296
rs137901416
rs6659669
rs871134
rs13230646
rs45604339
rs12932179
rs3767952
rs6881568
rs4743037
rs611646
rs117407747
rs1907702
rs11085072
rs17803849
rs7790856
rs1985369
rs7705526
rs8102497
rs932002
rs762679
rs34550383
rs66731853
rs76666449
rs7221585
rs79977579
rs2282764
rs6007020
rs112394943
rs28577594
rs1957937
rs2056726
rs10845387
rs12369950
rs11584821
rs10805346
rs141214782
rs10840270
rs4530278
rs73581419
rs11769630
rs939916
rs869785
rs12412214
rs10112752
rs78491606
rs10768683
rs61748181
rs2555104
rs9398196
rs142426306
rs116863223
rs1003322
rs2977608
rs376641875
rs11212631
rs56061761
rs10774624
rs7099229
rs3093888
rs41269079
rs113525195
rs111527438
rs2538745
rs4616688
rs7209057
rs9878436
rs6590343
All − MR Egger
All − Inverse variance weighted
−2
−1
0
1
2
MR effect size for
'telomere length || id:ieu-b-4879' on 'IDP_dMRI_TBSS_L2_Cingulum_cingulate_gyrus_
